# Characterization of intragenic tandem duplication in the *PAFAH1B1* gene leading to isolated lissencephaly sequence

**DOI:** 10.1186/s13039-015-0186-8

**Published:** 2015-10-31

**Authors:** Satoru Takahashi, Ryosuke Tanaka, Satomi Okano, Akie Okayama, Nao Suzuki, Hiroshi Azuma

**Affiliations:** Department of Pediatrics, Asahikawa Medical University, 2-1-1-1 Midorigaoka-Higashi, Asahikawa, Hokkaido 078-8510 Japan

**Keywords:** Duplication, Lissencephaly, Microhomology, Neuronal migration, *PAFAH1B1*

## Abstract

**Background:**

Genetic aberrations in *PAFAH1B1* result in isolated lissencephaly sequence (ILS), a neuronal migration disorder associated with severe mental retardation and intractable epilepsy. Approximately 60 % of patients with ILS show a 17p13.3 deletion or an intragenic variation of *PAFAH1B1* that can be identified by fluorescence in situ hybridization (FISH) analysis or gene sequencing. Using multiplex ligation-dependent probe amplification (MLPA), 40–80 % of the remaining patients show small genomic deletions or duplications of *PAFAH1B1*. The intragenic duplications within *PAFAH1B1* are predicted to abolish the PAFAH1B1 function, although a detailed characterization of the duplication regions have not been reported.

**Results:**

Here we describe a female patient with ILS occurring predominantly in the posterior brain regions. MLPA was used to identify a small duplication within *PAFAH1B1*. This result was confirmed by array-based comparative genomic hybridization analysis, revealing a duplication of the 29-kb region encompassing putative regulatory elements and exon 2 of *PAFAH1B1*. The region was characterized as an intragenic tandem duplication by sequencing, revealing a 28-bp microhomology sequence at the breakpoint junctions. Parental genetic testing confirmed that the tandem duplication occurred *de novo*. Reverse transcription-PCR on RNA extracted from peripheral blood leukocytes revealed that the expression level of *PAFAH1B1* decreased to that in a patient with Miller-Dieker syndrome, a contiguous gene-deletion disorder characterized by classical lissencephaly and a facial dysmorphism.

**Conclusions:**

This study expanded the spectrum of *PAFAH1B1* variants and identified a unique genomic architecture including microhomology sequences in *PAFAH1B1* underlying an intragenic tandem duplication leading to ILS.

## Background

Classical lissencephaly is a brain malformation resulting from impaired neuronal migration. The condition is characterized by absent (agyria) or decreased (pachygyria) convolutions of the cerebral cortex, causing the brain surface to appear smooth. Lissencephaly occurs either as part of Miller–Dieker syndrome (MDS), a multiple malformation syndrome with severe grade lissencephaly and a characteristic facial dysmorphism, or as an isolated condition without peculiar facial features termed isolated lissencephaly sequence (ILS) [1]. Most cases of ILS are caused by variations in either the *PAFAH1B1* gene on chromosome 17p13.3 or the *DCX* gene on chromosome Xp22.3 [2, 3]. *PAFAH1B1* variations result in more severe abnormalities in the posterior brain regions, whereas *DCX* variations cause more severe abnormalities in the anterior brain regions [4, 5]. MDS is caused by a deletion of genes in chromosome 17p13.3, which at minimum includes the *PAFAH1B1* and *YWHAE* genes [6].

The *PAFAH1B1* gene (also known as *LIS1*) spans approximately 92-kb and contains 11 exons, the first of which is noncoding. The cDNA contains a 1230-bp-long coding sequence and encodes a 410-amino-acid polypeptide with a calculated molecular mass of 45 kDa. PAFAH1B1 participates in microtubule-dependent cell motility by controlling the function of the cytoplasmic dynein motor protein and plays an important role in neuronal migration during brain development [7].

Here, we describe a case of a Japanese patient with ILS who had an intragenic tandem duplication of the region encompassing the putative regulatory elements and exon 2 of the *PAFAH1B1* gene. We characterized the boundary of the duplication region and found that each of the duplicated regions was flanked by an identical 28-bp sequence. The 28-bp microhomology potentially represents a molecular mechanism underlying the tandem duplication.

## Case presentation

### Case report

**Fig. 1:**
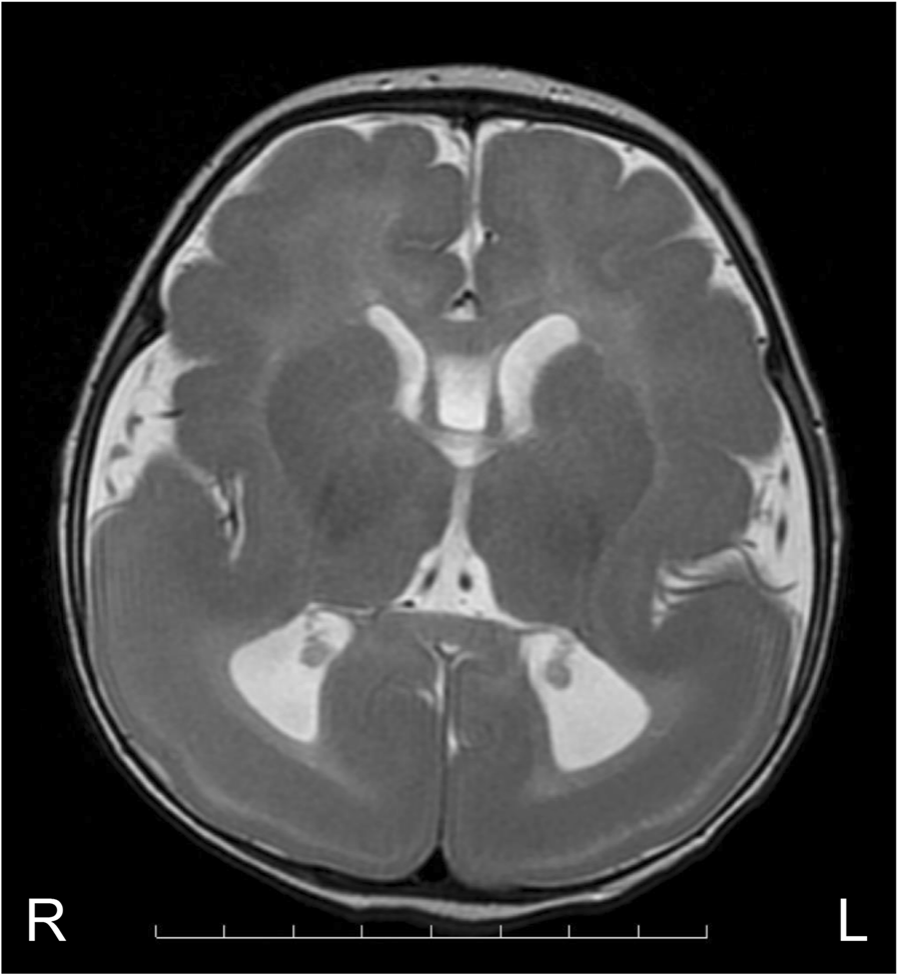
Brain MRI scan of a patient with an intragenic tandem duplication in the *PAFAH1B1* gene. Axial T2-weighted brain image indicating the occurrence of predominant agyria in the posterior regions, and pachygyria in the anterior regions. The patient was diagnosed with grade 3 lissencephaly

The patient, 4 years old at the time of this writing, is a Japanese female and the first child of nonconsanguineous parents. She was born at 40 weeks of gestation after an uneventful pregnancy with a birth weight of 2780 g, a length of 46 cm, and a head circumference of 32 cm (50th percentile). At the age of 2 months, she developed her first clonic seizures in the right arm. The tonic or clonic seizures also occurred in one or both limbs in the left side of her body. During the next 2 weeks, the polymorphous focal seizures became more frequent, occurring in clusters of 30–40 times a day. At the age of 3 months, she was referred to our hospital. Neurological examination revealed muscle hypotonia and a profound delay in psychomotor development. The patient had no eye contact and was not able to control her head. The hematological and chemical work-up was normal. Magnetic resonance imaging (MRI) of the brain demonstrated a thickened cortex and gyral malformations that were more severe in the posterior brain regions than in the anterior brain regions (Fig. 1). The patient did not show the characteristic facial appearance seen in patients with MDS. Therefore, on the basis of neuroradiological test results, she was diagnosed with ILS.

The patient’s interictal electroencephalography (EEG) results indicated low-amplitude background activity over the posterior head regions and multifocal spikes. Ictal recordings revealed focal, rhythmic theta activities originating from the right or left parietal regions and eventually spreading to the ipsilateral hemisphere.

Valproic acid treatment was initiated but was unsuccessful in controlling her seizures. In the next 2 weeks, the spasms appeared in clusters, and her interictal EEG results showed hypsarrhythmia. The patient’s condition was found to evolve to West syndrome. Treatment with synthetic adrenocorticotropic hormone (ACTH) was started wherein a single daily dose of 0.0125 mg/kg was administrated for 14 consecutive days, with subsequent tapering. The patient’s spasms ceased by day 6 of the ACTH therapy but reappeared during the withdrawal stage. Her psychomotor development was severely delayed without head control, eye pursuit, or the use of meaningful words. Because of feeding problems resulting from difficulties in swallowing, she was fed via a gastrostomy tube.

## Results

**Fig. 2:**
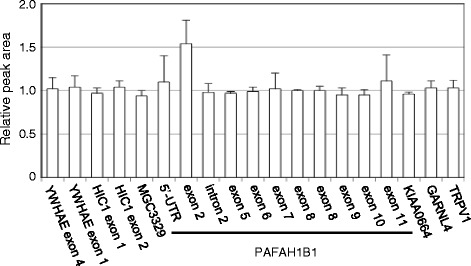
MLPA analysis performed on DNA from the patient revealed a small duplication in *PAFAH1B1* that included exon 2. Results indicate the relative peak area of a probe target sequence with normalization against normal female samples and are shown as the means ± SD (*n* = 3)

**Fig. 3:**
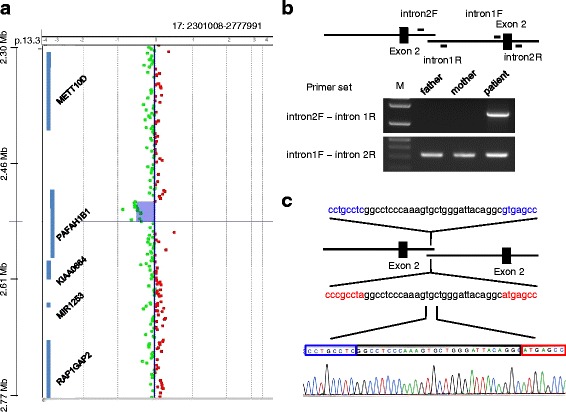
Intragenic tandem duplication in the *PAFAH1B1* gene in a patient with isolated lissencephaly sequence. **a** Array-CGH results showing the log2 intensity ratios of the patient versus reference DNA. A 29-kb duplication (purple-shaded region with green dots) was detected within the *PAFAH1B1* gene on chromosome 17p13.3. **b** Schematic representation of the duplicated region with the primer-binding sites. Long-range PCR was performed with the intron2F and intron1R primers, which yielded a 1569-bp PCR product from the patient’s genomic DNA, but not from her parents’ genomic DNA. These results indicated that the tandem duplication occurred *de novo*. **c** Sequencing analysis of the PCR product revealed region of identical sequences (28 nucleotides enclosed by black line) at the duplication junctions without additional sequence changes

The patient exhibited an abnormal gyral pattern characterized by agyria in the posterior brain regions and pachygyria in the anterior brain regions. These characteristics are highly suggestive of *PAFAH1B1* gene variations [4]. Fluorescence in situ hybridization (FISH) results for the 17p13.3 region and direct sequencing of the *PAFAH1B1* gene revealed no abnormalities in this patient. Therefore, we analyzed the patient’s *PAFAH1B1* gene via multiplex ligation-dependent probe amplification (MLPA) and identified a duplication of the region encompassing exon 2, but not exon 1 or 3 (Fig. 2). This result was confirmed by array-based comparative genomic hybridization (array CGH) analysis. The size of the duplication region was approximately 29 kb, spanning positions chr17:2,513,432 to 2,539,559 (GRCh37/hg19; Fig. 3a). To define the boundary of the duplication region, long-range polymerase chain reaction (PCR) was performed based on the array CGH and MLPA results. PCR performed using a forward primer in intron 2 and a reverse primer in intron 1 resulted in the amplification of a 1569-bp PCR product from the patient’s genomic DNA, but not from her parents’ genomic DNA (Fig. 3b), confirming that the duplication was intragenic and in tandem. These results further indicated that the duplication occurred *de novo* in the patient. Sequencing analysis of the PCR product revealed that each of the duplicated regions was flanked by an identical 28-bp sequence, indicating that the 28-bp microhomology was used to generate the tandem duplication (Fig. 3c).

**Fig. 4:**
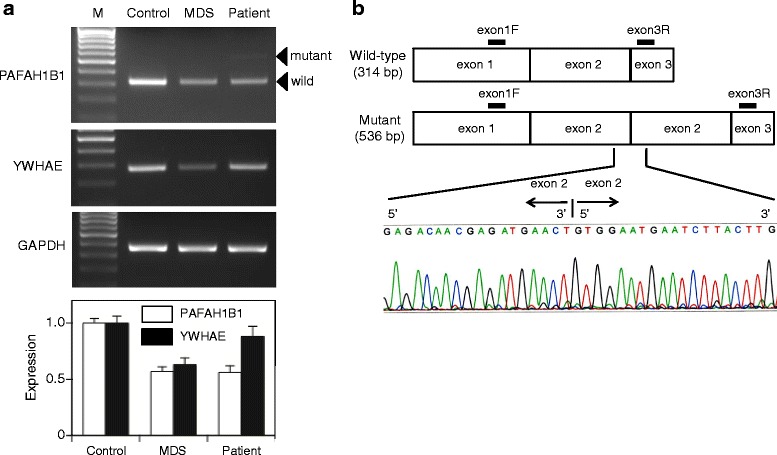
Decreased expression of *PAFAH1B1* in a patient with isolated lissencephaly sequence (ILS). **a** RT-PCR on RNA extracted from peripheral blood leukocytes revealed that the expression levels of *PAFAH1B1*, but not *YWHAE*, were decreased in this patient, while the expression levels of both the genes were decreased in a patient with Miller-Dieker syndrome (MDS). Note that a longer *PAFAH1B1* mRNA (536 bp) was present in the patient with ILS at a low level along with the normal mRNA (314 bp). The quantitative results in the bar graph indicate *PAFAH1B1* and *YWHAE* mRNA expression levels, relative to those of controls, and are shown as the mean ± SD (*n* = 3). Data are expressed in arbitrary units. **b** Sequencing analysis of the longer *PAFAH1B1* transcript found in the patient confirmed the tandem duplication of exon 2

Reverse transcription (RT)-PCR amplification with a forward primer in exon 1 and a reverse primer in exon 3 yielded two fragments with different lengths of 314-bp and 536-bp from the RNA of the patient, but only a 314-bp fragment from the control (Fig. 4a). Sequencing analysis of the novel 536-bp fragment confirmed that the transcript contained a tandem duplication of exon 2 (Fig. 4b). The *PAFAH1B1* variant showed low-level expression (Fig. 4a). The expression level of the normal *PAFAH1B1* transcript in the patient decreased to that in a patient with MDS (Fig. 4a).

## Discussion

Previous studies using MLPA have identified 30 deletions and 7 duplications within the *PAFAH1B1* gene among 57 patients with ILS occurring predominantly in the posterior brain regions, in whom FISH for 17p13.3 and gene sequencing gave negative results [8, 9]. These intragenic duplications are predicted to abolish the PAFAH1B1 function, although a detailed characterization of the duplication regions has not been reported. Here, we report our findings for a patient with posteriorly dominant ILS who had an intragenic tandem duplication in the *PAFAH1B1* gene. The *PAFAH1B1* expression level decreased to that in a patient with MDS. Analysis of the duplication boundary revealed a microhomology at the breakpoint, which suggested that the duplication might have been mediated in part by the microhomology sequence.

A recent report described the presence of microhomology sequences ranging from 2 to 75-bp in 79 % of the breakpoint junctions of rare pathogenic copy number variations (CNVs) [10]. These results indicate that rare pathogenic CNVs are caused by a replication-based mechanism of mitotic origin mediated by microhomology sequences in the context of local genomic architectures. Our investigation of the breakpoint region of the intragenic tandem duplication revealed a 28-bp microhomology sequence in introns 1 and 2 of the *PAFAH1B1* gene. In addition, the observation of no additional sequence changes at the junction indicates that the duplication resulted from a single replication-based event. Genetic testing of the patient’s parents confirmed that the tandem duplication variant occurred *de novo*. Thus, a single event of microhomology-mediated rearrangement might be the potential molecular mechanism whereby the tandem duplication variant occurred.

A gradient of severity in lissencephaly can aid the planning of appropriate genetic testing. ILS occurring predominantly in the posterior brain regions is most frequently associated with *PAFAH1B1* variants, but rarely with variants of the *TUBA1A* gene encoding tubulin alpha-1a [11]. We did not analyze the *TUBA1A* gene because the patient showed no additional malformations, such as cerebellar hypoplasia or the absence of the corpus callosum, which have been found in patients with *TUBA1A*-associated lissencephaly [11]. The duplication region identified in this patient includes putative noncoding regulatory elements of *PAFAH1B1* and its second exon, which harbors the ATG translational start site. The mutant mRNA containing the tandem exon 2 duplication showed low-level expression. The 5′-untranslated region (5′-UTR) contains a variety of cis-regulatory elements which influences the expression of the downstream genes. Hence, this duplication potentially affected *PAFAH1B1* expression through destabilizing the secondary structure of the 5′-UTR. A balanced chromosomal translocation that disrupted the 5′-UTR of the *PAFAH1B1* gene has been found in a patient with lissencephaly [12]. The heterozygous deletion of *YWHAE*, together with *PAFAH1B1* in patients with MDS, is known to increase the severity, usually with grade 1 lissencephaly (diffuse agyria) being observed [6]. The patient in this study showed decreased expression of *PAFAH1B1*, but not *YWHAE*, resulting in grade 3 lissencephaly (mixed agyria and pachygyria).

## Conclusions

Our findings expanded the spectrum of *PAFAH1B1* variants implicating in ILS. We observed a heterozygous intragenic tandem duplication involving the putative noncoding regulatory elements and exon 2 of *PAFAH1B1*, which resulted in decreased expression of this gene in a patient with ILS. We further found a unique genomic architecture including microhomology sequences in introns 1 and 2 of the *PAFAH1B1* gene underlying an intragenic tandem duplication leading to ILS.

## Methods

### Mutation screening

This study was performed in compliance with the guidelines of Asahikawa Medical University. After obtaining written informed consent from her parents, genomic DNA was extracted from the peripheral blood leukocytes of both the patient and her parents, and was used for variant screening. Appropriate primers were used to yield DNA fragments spanning the entire coding region and intron–exon boundaries of *PAFAH1B1* [5]. The PCR fragments were analyzed by automated sequencing. Gene dosage analysis was performed using the SALSA MLPA Kit P061-B1 Lissencephaly (MRC-Holland, Amsterdam, The Netherlands). To define the boundary of the duplicated region, array CGH analysis was performed using a high-resolution 1 M array (Agilent Technologies Inc.; Santa Clara, CA).

### Long-range PCR amplification and DNA sequencing analysis

Long-range PCR was performed using TaKaRa LA Taq Polymerase (TaKaRa Bio Inc.; Otsu, Japan). Primers were designed for amplification and sequencing of the duplication junction within *PAFAH1B1* including a forward primer in intron 2 (intron2F; 5′-GTGTGGAAGACACTTAGTGGC-3′) and a reverse primer in intron 1 (intron1R; 5′-GGCCTACATCCTGACTTGAC-3′). To verify the DNA quality for PCR amplification, the following primers for amplification of *PAFAH1B1* exon 2 were also used: a forward primer in intron 1 (intron1F; 5′- TGTGGAAGACACTTAGTGGCA-3′) and a reverse primer in intron 2 (intron2R; 5′- AAGAGACCTCCCAAAGCTGTA-3′), which generated a 269-bp product [5]. The obtained PCR products were directly sequenced on an ABI 3130 Genetic Analyzer (Applied Biosystems; Foster City, CA).

### RNA isolation and RT-PCR

Total RNA was extracted from the peripheral blood leukocytes using the PAXgene Blood RNA Kit (Qiagen GmbH; Hilden, Germany). The RT step was performed using the SuperScript First-Strand Synthesis System (Invitrogen Corporation; Carlsbad, CA) to generate cDNA by using 1 μg of total RNA in a 20-μl reaction. The following primers were used: for *PAFAH1B1*, 5′-GAGTGAAGGACGGAAGAGGC-3′ and 5′-GAATATGCCTCTTCATAGCC-3′, generating a 314-bp product; for *YWHAE*, 5′-ACGACGAAATGGTGGAGT-3′ and 5′-AGCTGCTGGAATGAGGTG-3′, generating a 278-bp product; and for *GAPDH*, 5′-CCAGCCGAGCCACATCGCTC-3′ and 5′-ATGAGCCCCAGCCTTCTCCAT-3′, generating a 360-bp product. The PCR products were visualized by ethidium bromide staining, following electrophoresis on 2 % agarose gels. The optical densities of the bands were quantified using an image analysis system and ImageJ software (National Institutes of Health; Bethesda, MD). Selected RT-PCR products were subsequently cloned into the pCR4-TOPO vector (Invitrogen Corporation; Carlsbad, CA) for sequencing.

## Consent

Written informed consent was obtained from the parents of the patients for publication of this case report. A copy of the written consent is avaible by the Editor-in –chief of this journal.

## Abbreviations

ACTH: adrenocorticotropic hormone
CGH: comparative genomic hybridization
CNV: copy number variation
DCX: doublecortin
EEG: electroencephalography
FISH: fluorescence in situ hybridization
ILS: isolated lissencephaly sequence
MDS: Miller–Dieker syndrome
MLPA: multiplex ligation-dependent probe amplification
MRI: magnetic resonance imaging
PAFAH1B1: platelet-activating factor acetylhydrolase 1b, regulatory subunit 1
PCR: polymerase chain reaction
RT: reverse transcription
TUBA1A: tubulin alpha-1a
UTR: untranslated region
YWHAE: tyrosine 3-monooxygenase/tryptophan 5-monooxygenase activation protein, epsilon
